# Chemical Genetics Applied to Elucidate the Physiological Role of Stress-Signaling Molecules on the Wound-Induced Accumulation of Glucosinolates in Broccoli

**DOI:** 10.3390/plants10122660

**Published:** 2021-12-03

**Authors:** Ana M. Torres-Contreras, Vimal Nair, Carolina Senés-Guerrero, Adriana Pacheco, Mauricio González-Agüero, Perla A. Ramos-Parra, Luis Cisneros-Zevallos, Daniel A. Jacobo-Velázquez

**Affiliations:** 1 Tecnologico de Monterrey, Escuela de Ingeniería y Ciencias, Av. Eugenio Garza Sada 2501, Monterrey C.P. 64849, Nuevo Leon, Mexico; marieltorres2811@gmail.com (A.M.T.-C.); adrianap@tec.mx (A.P.); perlaramos@tec.mx (P.A.R.-P.); 2 Department of Horticultural Sciences, Texas A&M University, College Station, TX 77843, USA; vimal.nair16@gmail.com (V.N.); lcisnero@tamu.edu (L.C.-Z.); 3 Tecnologico de Monterrey, Escuela de Ingeniería y Ciencias, Av. General Ramón Corona 2514, Zapopan C.P. 45201, Jalisco, Mexico; carolina.senes@tec.mx; 4 Postharvest Unit, Institute for Agricultural Research, INIA-La Platina, Santa Rosa, Santiago 11610, Chile; maugonza@gmail.com

**Keywords:** stress-signaling pathways, wounding stress, glucosinolate biosynthesis, chemical-genetic approach, secondary metabolism

## Abstract

Wounding stress is an effective strategy to induce glucosinolate (GS) biosynthesis in broccoli. However, there is insufficient knowledge on the physiological and molecular mechanisms underlying this stress response. Herein, a chemical-genetic approach was applied to elucidate the role of jasmonic acid (JA), ethylene (ET), and reactive oxygen species (ROS) on the wound-induced biosynthesis of GS. Broccoli was processed into chops to induce wounding stress. Broccoli chops were treated with phenidone (PHEN) and diphenyleneiodonium chloride (DPI) as inhibitors of JA and ROS biosynthesis, respectively, whereas 1-methylcyclopropene (1-MCP) was applied as an inhibitor of ET action. Wounding stress induced the expression of genes related to the biosynthesis of indolic and aliphatic GS, which was correlated with the accumulation of GS and modulated by the inhibitors of signaling molecules applied. Results of gene expression analysis indicated that JA played a key role in the activation of most genes, followed by ROS. Furthermore, except for the *CYP79B2* gene, PHEN and 1-MCP synergistically downregulated the expression of GS biosynthetic genes evaluated, showing that the interaction between JA and ET was fundamental to modulate GS biosynthesis. Results presented herein increased our knowledge of the physiological and molecular mechanisms governing the wound-induced biosynthesis of GS in broccoli.

## 1. Introduction

The wound-induced biosynthesis of glucosinolates (GS) has been previously reported for broccoli [1,2]. However, little is known on the physiological and molecular mechanisms governing this stress response. Recently, a transcriptome analysis by RNA-Seq revealed that postharvest wounding stress in broccoli induces a genetic response, which is dependent on the wounding intensity applied and the time elapsed after the application of the stress [3]. Metabolic pathways associated with the primary metabolism, including glycolysis, tricarboxylic acid cycle, and amino acid biosynthesis, were highly up-regulated due to wounding stress. Likewise, jasmonic acid (JA), and ethylene (ET) biosynthesis, as well as the shikimate, phenylpropanoid and glucosinolate pathways were also highly induced by wounding stress [3]. These results set the groundwork for the elucidation of the physiological response that generates the wound-induced biosynthesis and accumulation of GS in broccoli. However, there is still a lack of information at the physiological and molecular level regarding the role of stress-signaling molecules on the wound-induced accumulation of GS in broccoli.

Plant hormones such as JA and ET have a crucial role in plant stress responses since they are signaling molecules that induce the biosynthesis and accumulation of secondary metabolites including GS [3,4,5,6]. Additionally, reactive oxygen species (ROS) have been identified as key signaling molecules that induce secondary metabolite biosynthesis in different crops [6,7,8]. The availability of small molecules that inhibit the biosynthesis or action of these signaling molecules allows the application of chemical genetics approaches to elucidate the role of signaling pathways in certain physiological processes in plant tissues [9]. For instance, 1-methylcyclopropene (1-MCP) can be applied as inhibitor of ET action, whereas phenidone (PHEN) and diphenyleneiodonium chloride (DPI) can be applied as inhibitors for the biosynthesis of JA and ROS, respectively [7,8]. The efficacy of these signaling-molecule inhibitors (PHEN, DPI, and 1-MCP) has been previously documented and applied in chemical genetic studies [10,11,12,13,14]. For instance, DPI has effectively inhibited ROS production mediated by NADPH oxidase in *Arabidopsis* [10]. Similarly, PHEN has inhibited lipoxygenase activity and its corresponding JA accumulation in plants from *Acacia* species [11] and *Zea mays* [12]. Finally, the inhibition of ET perception exerted by 1-MCP has been well documented for several fruits and vegetables [13,14]. 

In the last decade, the role of JA, ET, and ROS on the postharvest wound-induced biosynthesis of phenolics has been extensively studied in carrot [7]. The first insight was the proposal of a crosstalk signaling model among ROS, ET, and JA that governs the wound response in carrots [7]. In this model, ROS play a key role as modulators for the wound-induced activation of the primary and secondary metabolisms in carrot, while ET and JA regulate the concentration of ROS in the tissue and exert direct activities on the primary and secondary metabolism as well.

The aim of this study was to increase our knowledge on the physiological role of JA, ET, and ROS on the wound-induced biosynthesis and accumulation of GS in broccoli. To achieve this objective, a chemical genetic approach was performed, using specific inhibitors of stress-signaling pathways and measuring their effects on the expression of GS biosynthetic genes and on the accumulation of total and individual GS. Genes studied included *CYP79B2*, the sulfotransferases *ST5a*, *ST5b*, and *ST5c*, the transcription factor *MYB122*, and the *IGMT1* gene, previously reported to be highly wound-induced in broccoli [3]. *CYP79B2* gene product catalyzes the first step in the indolic glucosinolate biosynthesis pathway [15]. Sulfotransferases (ST) catalyze the last stage of glucosinolate core structure biosynthesis [16], whereas the transcription factor *MYB122* is known to regulate indolic glucosinolate biosynthesis [17]. Finally, *IGMT1* gene code for indole glucosinolate *O*-methyltransferase 1, the enzyme that catalyzes indole GS modification to generate the final structures [18].

## 2. Results and Discussion

To better understand the role of JA, ET, and ROS on the wound-induced activation of the glucosinolate biosynthesis pathway, a chemical genetics approach was followed, where PHEN, 1-MCP, and DPI were used as inhibitors of JA, ET, and ROS, respectively. The sampling time (21 h) to quantify total and individual glucosinolates was determined according to a preliminary study shown in Supplementary Materials (Table S1). Furthermore, the sampling time to evaluate the expression of GS biosynthetic genes (9 h) was selected based on a previously reported transcriptome analysis, where the differential expression of secondary metabolism-related genes was determined as an early and late response to wounding stress in broccoli [3].

To determine the effect of signaling molecule inhibitors on the expression of genes and on the accumulation of total and individual GS, two controls were used: chopped broccoli and chopped broccoli dipped into distilled water. Chopped broccoli non-dipped in water was used as the control to evaluate the effect of 1-MCP when applied alone, whereas chopped broccoli dipped in water was used as the control for samples treated with PHEN or DPI either applied alone or in combination with each other and with 1-MCP.

### 2.1. Effect of Signaling Molecule Inhibitor Concentrations on Glucosinolate (GS) Accumulation

Individual glucosinolates identified in broccoli are shown in Supplementary Materials, Figure S1. To determine the optimum concentration of signaling molecules to block the wound-induced biosynthesis of GS in broccoli chops, they were treated with three different concentrations of inhibitors DPI (3.17, 31.7, and 317 μM), PHEN (0.1, 1.0, and 10 mM), and 1-MCP (500, 1000, and 2000 ppb). After, samples were stored at 20 °C for 21 h, and the concentration of GS was determined. Control samples showed wound-induced accumulation of glucobrassicin, 4-hydroxyglucobrassicin, 4-metoxyglucobrassicin, and glucoraphanin after storage (Table 1). Dipping in water resulted in the leaching of glucoraphanin and glucobrassicin into water since their concentration in water control samples was lower than in control samples at time 0 h.

Interestingly, after storage, 4-metoxyglucobrassicin presented a higher increase in water control than in the control. Glucoraphanin content decreased with the highest concentration of 1-MCP (2000 ppb) but increased when dipped in the highest concentration of DPI (317 μM) or PHEN (10 mM) and even when exposed to 1000 ppb of 1-MCP as compared with their corresponding control samples (Table 1). Regarding glucobrassicin, its content was not affected by DPI at any concentration, while the use of 1-MCP at the highest concentration caused a significant decrease. The three PHEN concentrations also caused a significant decrease and were shown to be dose-dependent, since the higher PHEN concentration, the lower glucobrassicin content in the samples. The 4-metoxyglucobrassicin content decreased by using the intermediate concentration of 1-MCP (1000 ppb) and PHEN (1.0 mM). DPI showed dose-dependent response in 4-metoxyglucobrassicin content since it decreased as the concentration of DPI increased (Table 1). Finally, the same trend was observed in the three inhibitors for 4-hydroxyglucobrassicin: the higher inhibitor concentration, the lower content of the compound in samples. The highest concentration of 1-MCP (2000 ppb), DPI (317 μM), and PHEN (10 mM) decreased the content of 4-hydroxyglucobrassicin by 74, 75, and 40%, respectively, as compared to their corresponding control samples (Table 1). The application of inhibitors affected each glucosinolate differently. According to the results, the highest dose of each inhibitor was selected (DPI, 317 μM; 1-MCP, 2000 ppb; PHEN, 10 mM) to evaluate their individual and combined effect on the expression of glucosinolate biosynthetic genes and the accumulation of individual glucosinolates.

### 2.2. Role of JA, ET, and ROS on the Wound-Induced Activation of Glucosinolate Biosynthetic Genes 

**Figure 1:**
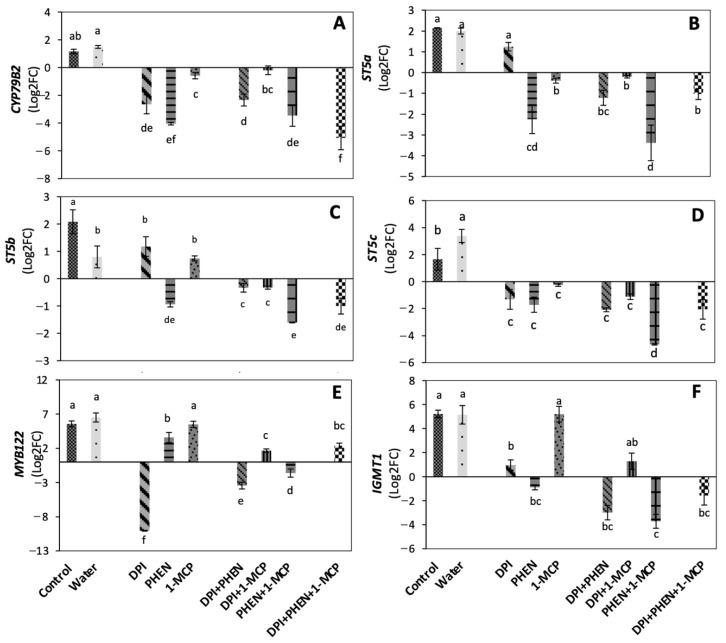
Effect of stress-signaling molecule inhibitors on the relative expression of glucosinolate biosynthetic genes. *CYP79B2* (**A**); *ST5a* (**B**); *ST5b* (**C**); *ST5c* (**D**); *MYB122* (**E**); *IGMT1* (**F**). Relative expression is shown at 9 h after wounding. Expression was normalized with the house-keeping gene *actin 2* and relative to time 0 h samples. Values represent the mean of three replicates ± standard error of the mean. Bars with different letters (a, b, c, d, e, and f) indicate significant difference by the LSD test (*p* < 0.05). Abbreviations: diphenyleneiodonium chloride (DPI), phenidone (PHEN), 1-methylcyclopropene (1-MCP), *cytochrome P450* gene (*CYP79B2*), *sulfotransferase a* (*STSa*), *sulfotransferase b* (*STSb*), *sulfotransferase c* (*STSc*), *MYB transcription factor* gene (*MYB122*), *indole glucosinolate O-methyltransferase 1* (*IGMT1*).

The effect of individual and combined application of PHEN, 1-MCP, and DPI on the relative expression of genes related with the biosynthesis of GS is shown in Figure 1. *CYP79B2* gene product catalyzes the first step in the indolic glucosinolate biosynthesis pathway [15]. Wounding induced a slight increase in the relative expression of *CYP79B2* gene (normalized with the house-keeping gene *actin 2* and relative to time 0 h samples), while the use of individual inhibitors alone or combined impeded its wound-induced expression (Figure 1A).

The use of PHEN and DPI applied individually caused a high repression in the expression of *CYP79B2* gene, suggesting that JA and ROS play a key role in the wound-induced activation of indolic glucosinolate biosynthesis, whereas ET plays a slight role. These observations agree with previous findings in *Brassica* species [15,19,20]. For instance, in *Arabidopsis*, *CYP79B2* was reported to be inducible by wounding and by MeJA treatment [15]. Likewise, in broccoli cell culture, *CYP79B2* was reported to be highly induced by coronatine treatment (mimicking JA action) and was closely correlated with an increase in GS content [19]. Furthermore, 1-MCP application during postharvest of broccoli was reported to reduce *CYP79B2* gene expression during storage [20]. Since *CYP79B2* was shown to be responsible for the de novo indolic glucosinolate biosynthesis during postharvest storage; the results indicate that JA, ET, and ROS are involved in the de novo wound-induced biosynthesis of indolic GS.

Sulfotransferases (ST) catalyze the last step of glucosinolate core structure biosynthesis [16]. The three sulfotransferases (*ST5a*, *ST5b*, *ST5c*) were shown to be induced by wounding (Figure 1B–D). Dipping in water decreased the induction of *ST5b* (Figure 1C) but increased the expression of *ST5c* (Figure 1D). The application of DPI did not affect the relative expression of *ST5a* and *ST5b*, while it repressed the wound-induced activation of *ST5c*. PHEN decreased the expression of the three sulfotransferases genes, while 1-MCP affected *ST5a* and *ST5c* expression, but not *ST5b*. Additionally, when PHEN was used in combination with 1-MCP, an additive effect in decreasing sulfotransferase expression was observed. These results agree with previous reports where sulfotransferases were reported to be differentially expressed under various conditions in *Arabidopsis* [21,22]. In a broccoli cell culture, *ST5a* was reported to be highly induced by a coronatine treatment and closely associated with higher glucobrassicin production [19]. Since each sulfotransferase has been reported to have substrate specificity, these differences in expression patterns as response to signaling-molecule inhibitors indicate that JA, ET, and ROS are responsible for modulating the GS profile of broccoli under wounding stress.

The transcription factor *MYB122* is known to regulate indolic glucosinolate biosynthesis [17]. *MYB122* wound-induction was highly repressed by DPI application and slightly down-regulated by the use of PHEN, whereas 1-MCP application did not affect the expression of the gene (Figure 1E). This result is opposite to what was reported for the induction of *MYB122* triggered by copper ions, where the ET signaling transduction pathway was required to induce the response [23]. The results presented herein showed that wounding can induce *MYB122* without ET signal, whereas ROS play a major role in its upregulation. Likewise, when PHEN was combined with 1-MCP or DPI, a repression of the *MYB122* relative expression was observed. Interestingly, the application of DPI combined with 1-MCP or the three inhibitors together caused a slight induction of the gene when compared with the control samples, suggesting a complex cross-talk among the signaling molecules that induce *MYB122* expression. Our results suggest that ROS play a major role in the wound-induced expression of *MYB122*, while JA and ET play a secondary role. This observation agrees with a previous report showing that *MYB122* plays a minor role in JA/ET-induced glucosinolate biosynthesis [17].

*IGMT1* gene code for indole glucosinolate *O*-methyltransferase 1, which catalyzes the modification of indole GS to generate the final structures [18]. The wound-induced expression of *IGMT1* decreased by the individual application of DPI and PHEN, but no effect was observed when 1-MCP was applied alone (Figure 1F). Furthermore, the combination of DPI+PHEN and PHEN+1-MCP significantly reduced *IGMT1* gene expression, as well as the combination of the three inhibitors. These results indicate that ROS and JA play a major role in the activation of *IGMT1*. Moreover, a crosstalk between JA and ET on the wound-induced activation of *IGMT* was also demonstrated.

### 2.3. Role of JA, ET, and ROS on the Wound-Induced Accumulation of Glucosinolates 

#### 2.3.1. Total Glucosinolate Accumulation

**Figure 2:**
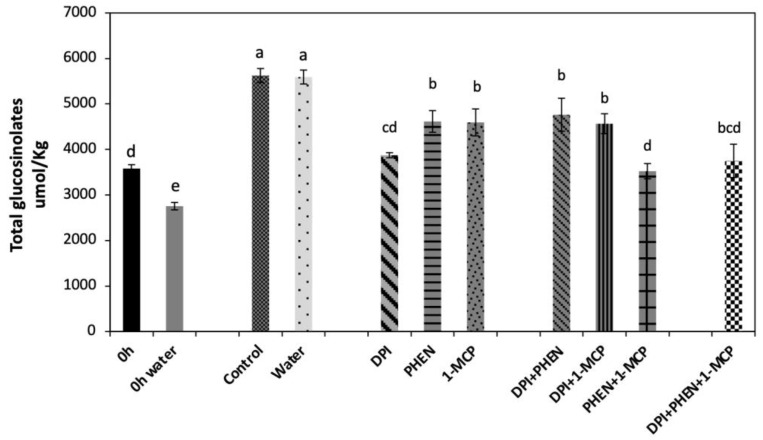
Effect of stress-signaling molecule inhibitors on the wound-induced accumulation of total glucosinolates in broccoli. Glucosinolates were quantified in broccoli chops before and after 21 h of storage. Values represent the mean of three replicates ± standard error of the mean. Concentrations are reported on a dry weight basis. Bars with different letters (a, b, c, d, and e) indicate statistical difference by the LSD test (*p* < 0.05). Abbreviations: diphenyleneiodonium chloride (DPI), phenidone (PHEN), and 1-methylcyclopropene.

Total glucosinolate content was calculated as the sum of individual GS quantified, which were composed of indolic (48%) and aliphatic (52%) glucosinolates (Figure 2). As an immediate effect of dipping chopped broccoli in water, a significant decrease (−23%) in total GS was detected. This could be attributed to a leaching effect of GS from the broccoli tissue into water. This result was as expected due to the presence of glucose and a negative charge on the sulfate group of glucosinolate structure, resulting in a highly polar molecule [24]. After storage (21 h at 20 °C), both controls (chops and chops dipped in water) reached the same total GS content, which was 57% higher than the control before storage.

The application of 1-MCP produced a slight decrease (−18%) in the wound-induced accumulation of total GS as compared with the control (Figure 2). Likewise, PHEN application decreased GS accumulation at the same level of 1-MCP treatment, whereas the largest decrease (−32%) was observed in the DPI treatment. The use of DPI in combination with 1-MCP or PHEN slightly decreased (−16%) the wound-induced accumulation of GS. Moreover, combining PHEN with 1-MCP caused lower accumulation of GS (−37%) when compared with treatments of two inhibitors containing DPI. Therefore, results indicate that the signaling molecules evaluated in this study individually (ET, ROS, and JA) play a role in the wound-induced accumulation of GS in broccoli. Furthermore, JA and ET act synergistically to induce the biosynthesis of GS, which agree with the relative expression of the genes quantified (Figure 1). As previously described, the combined application of PHEN and 1-MCP highly repressed the wound-induced activation of glucosinolate biosynthesis-related genes. These results are also supported by previous reports, where the application of ET and/or methyl jasmonate (MeJA) induced a significant increase in the GS content in broccoli heads, florets, and wounded-florets [1], as well as on broccoli florets subjected to ultrasound treatment [25].

#### 2.3.2. Individual Glucosinolate Accumulation

**Figure 3:**
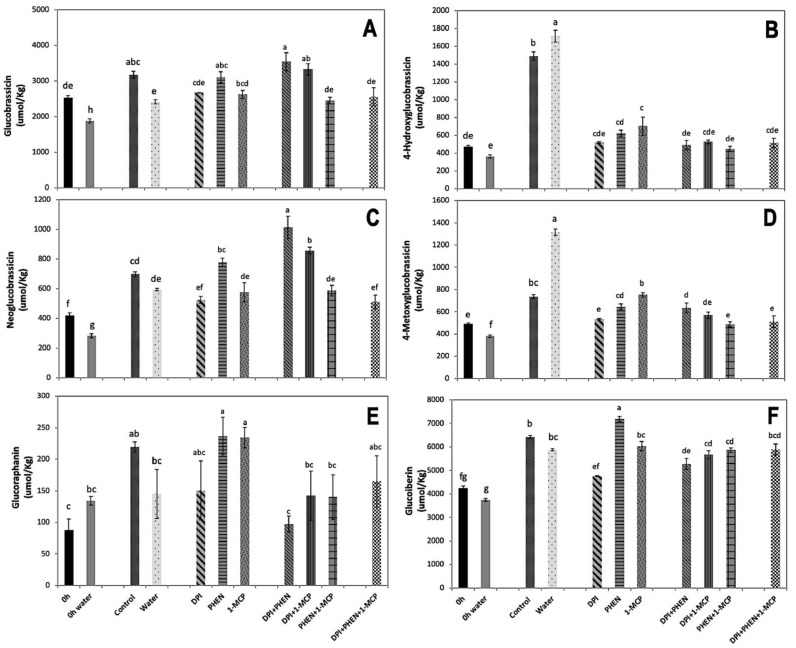
Effect of stress-signaling molecule inhibitors on the wound-induced accumulation of individual GS in broccoli. Glucobrassicin (**A**); 4-hydroxyglucobrassicin (**B**); neoglucobrassicin (**C**); 4-metoxyglucobrassicin (**D**); glucoraphanin (**E**); glucoiberin (**F**). Glucosinolates were quantified in broccoli chops before and after 21 h of storage. Values represent the mean of three replicates ± standard error of the mean. Concentrations are reported on a dry weight basis. Bars with different letters (a, b, c, d, e, f, and g) indicate statistical difference by the LSD test (*p* < 0.05). Abbreviations: diphenyleneiodonium chloride (DPI), phenidone (PHEN), and 1-methylcyclopropene (1-MCP).

The effect of signaling molecule inhibitors on the accumulation of individual indolic and aliphatic GS was evaluated (Figure 3). The individual use of DPI did not affect the accumulation of glucobrassicin and neoglucobrassicin, whereas PHEN application increased the accumulation of both GS as compared with the water control. The application of 1-MCP did not affect glucobrassicin accumulation (Figure 3A), while it slightly reduced the content of neoglucobrassicin (Figure 3C). Interestingly, the application of DPI combined with 1-MCP or PHEN resulted in a significant increase in the concentration of glucobrassicin and neoglucobrassicin as compared with the control. PHEN combined with 1-MCP and the use of the three inhibitors did not affect the wound-induced accumulation of glucobrassicin and neoglucobrassicin (Figure 3A,C).

On the other hand, 4-hydroxyglucobrassicin and 4-metoxyglucobrassicin, showed a similar response to inhibitors. These compounds showed wound-induced accumulation after 21 h of storage at 20 °C, which was enhanced by dipping chopped broccoli in water (Figure 3B,D). The application of PHEN, 1-MCP, or DPI individually and in combination caused a significant decrease in the accumulation of 4-hydroxyglucobrassicin and 4-metoxyglucobrassicin, practically impeding their accumulation (Figure 3B,D). This observation agrees with the expression of *CYP79B2* gene quantified (Figure 1A), which is involved in the first step of indolic glucosinolate biosynthesis [26].

The results presented herein are in contrast with previous reports, where the exogenous application of MeJA induced the accumulation of glucobrassicin and neoglucobrassicin biosynthesis in broccoli [27,28]. Likewise, Villarreal-García et al. [1] found that the postharvest exogenous application of ET or MeJA in wounded broccoli florets induced neoglucobrassicin accumulation. Since blocking JA biosynthesis in wounded broccoli did not reduce but rather increased the accumulation of these compounds, it is likely that ET and/or ROS are compensating the inhibitory effect of PHEN on the accumulation of the compounds. This result suggests that the wound-induced accumulation of neoglucobrassicin is the result of a complex crosstalk between the signaling molecules.

The decrease in the wound-induced accumulation of 4-hydroxyglucobrassicin and 4-metoxyglucobrassicin by using DPI, PHEN, or 1-MCP agrees with previous studies [1,20]. It was recently reported that the exogenous application of ET results in the accumulation of 4-hydroxyglucobrassicin content in broccoli [1]. On the other hand, the application of 1-MCP caused a decrease in 4-methoxyglucobrassicin in broccoli [20]. These authors proposed that the postharvest exogenous application of 1-MCP helps to maintain glucobrassicin concentration by the inhibition of the 4-methylation of glucobrassicin in broccoli [20]. The results presented herein correlate with those previous observations. The application of 1-MCP inhibited the wound-induced accumulation of 4-methoxyglucobrassicin while maintaining the biosynthesis of glucobrassicin. This result did not correlate with the expression of *IGMT1* gene detected on the tissue treated with inhibitors (Figure 2F), since 1-MCP did not affect its expression. Therefore, it is likely that ET is modulating other steps related with the accumulation of 4-methoxyglucobrassicin, either affecting the production of precursors or activating the transformation of glucosinolate into its corresponding isothiocyanate catalyzed by myrosinase, resulting in lower accumulation of the metabolite.

For each individual glucosinolate, the effect of blocking signaling molecules in the wound-response was shown to have a different effect over its biosynthesis and accumulation. Previously, it was reported that different molecules related with stress caused different response in the accumulation of individual GS [29,30]. Therefore, the results from the present study indicate that wounding stress activates a series of physiological and molecular responses that do not follow a single established pathway but rather a dynamic behavior, in which the conditions of the plant direct the carbon flux according to the availability of specific molecules and signals.

Regarding aliphatic GS, the content of glucoraphanin and glucoiberin increased in broccoli chops without inhibitors (Figure 3E,F). The use of inhibitors and its combinations did not affect the wound-induced accumulation of glucoraphanin, except for the application of PHEN alone, which produced a significant increment (65%). Likewise, glucoiberin also showed an increment (20%) when PHEN was used alone. The application of DPI alone and combined with PHEN caused a decrease in the wound-induced accumulation of glucoiberin, while the other treatments did not cause an effect (Figure 3F). The results indicate that for aliphatic glucosinolate biosynthesis, particularly glucoraphanin and glucoiberin, JA acts as inhibitor, since blocking its production allowed their accumulation (Figure 3E,F). This agrees with a previous report, where the exogenous application of methyl jasmonate in broccoli florets generated a decrease in glucoraphanin concentration [1]. This phenomenon can be attributed to the methyl jasmonate mediated up-regulation of myrosinase (*BoMyo*) and the myrosinase enzyme co-factor gene *epithiospecifier modifier1* (*BoESM1*), which according with a previous report correlated with the transformation of glucoraphanin to sulforaphane, impeding glucoraphanin accumulation [20].

**Figure 4:**
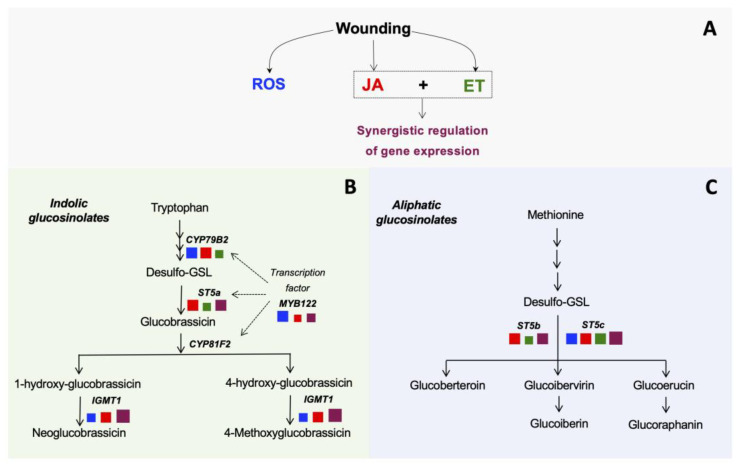
A scheme summarizing the role of stress-signaling molecules on the wound-induced activation of glucosinolate biosynthetic-related genes in broccoli. The model is focused on the role of jasmonic acid (JA), reactive oxygen species (ROS), and ethylene (ET), which are the signaling molecules herein evaluated. The production of signaling molecules occurs after wounding stress (**A**), which further upregulates the expression of genes involved in the biosynthesis of indolic (**B**) and aliphatic (**C**) glucosinolates. The color of the squares next to the gene name indicates the signaling molecule that upregulates its wound-induced gene expression. Blue, red, and green squares indicate upregulation by ROS, JA, and ET, respectively. Maroon squares indicate synergistic upregulation of the gene by JA and ET. The square size indicates the impact level of each signaling molecule on the wound-induced activation of the gene.

In summary, wounding stress in broccoli induced the expression of genes related with the biosynthesis of indolic and aliphatic GS. This wound-induced activation of genes was correlated with the accumulation of GS and modulated by the signaling molecules herein evaluated (JA, ET, and ROS). The role of each signaling molecule on the wound-induced activation of GS biosynthetic genes is summarized in Figure 4. According to previous reports, wounding induces the production of JA, ET, and ROS in broccoli [3,4]. JA was the signaling molecule that played a key role in most of the genes evaluated, followed by ROS. Regarding the interaction between stress-signaling molecules, except for the *CYP79B2* gene, JA and ET synergistically upregulated the expression of GS biosynthetic genes herein evaluated; thus, this interaction seems to be fundamental to modulate the wound-response in broccoli.

Further studies on understanding the role of signaling molecules on the wound-induced biosynthesis of GS in plants should consider including additional glucosinolate biosynthetic genes to validate the findings presented herein. Likewise, aromatic glucosinolates were not included in the study since their accumulation is not affected by wounding in broccoli [2,3]. However, other plant models rich in aromatic amino acids, such as *Arabidopsis*, could be used to understand better how wounding affects their biosynthesis and the underlying mechanism.

## 3. Materials and Methods

### 3.1. Chemicals

Desulfoglucoraphanin was purchased from Santa Cruz Biotechnology (Dallas, TX, USA). 1-methylcyclopropene (1-MCP) powder (SmartFreshTM) was kindly provided by AgroFresh Inc. (Springhouse, PA, USA). The rest of the chemicals were acquired from Sigma Chemical Co. (St. Louis, MO, USA).

### 3.2. Plant Material, Processing, and Storage of Broccoli Samples

Broccoli (*Brassica oleracea*) var. Heritage was harvested in Aguascalientes (Aguascalientes, Mexico) in September 2017. Before applying wounding stress, the plant material was disinfected with chlorinated water (200 ppm, pH 6.5) and stored for 12 h at 20 °C in an incubator (VWR, Radnor, PA, USA). To induce wounding stress, broccoli florets were processed into chops using a commercial food processor (Waring Commercial, WFP11, Torrington, CT, USA). Broccoli chops were stored in an incubator at 20 °C for 21 h.

### 3.3. Application of Stress-Signaling Molecule Inhibitors

Broccoli chops were treated with stress-signaling molecule inhibitors (PHEN, 0.1, 1.0, and 10 mM; DPI, 3.17, 31.7, and 317 μM; 1-MCP, 500, 1000, and 2000 ppb) to determine their optimum concentration to decrease the biosynthesis of GS. Once the optimum concentration was determined (PHEN 10 mM; DPI 317 μM; 1-MCP 2000 ppb), inhibitors were applied either individually or in combination in broccoli chops as previously described [7,8]. To inhibit ROS and JA production, chopped broccoli (200 g) were dipped in 500 mL of DPI or PHEN solution for 60 s, respectively. Finally, the excess water was drained using a plastic mesh. To obtain DPI and PHEN solutions, the inhibitors were dissolved in 0.1 mL DMSO (dimethyl sulfoxide) or 2% methanol, respectively, before dilution with distilled water (Milli-Q, Millipore Corp., Bedford, MA, USA) to reach the desired concentrations. To block ET action, whole broccoli heads were exposed to headspace vapors of 1-MCP for 12 h before wounding to block ET receptors before wounding, since ET production is immediately activated as a wound-response [7,8]. Application of 1-MCP (SmartFresh^TM^) was performed in a 20 L closed container, following instructions provided by AgroFresh, Inc. (Spring House, PA, USA). All treatments were performed at 20 °C.

To determine the effect of the inhibitors’ application on the expression of genes and on the accumulation of GS, two controls were used: chopped broccoli (referred to as control) and chopped broccoli dipped into distilled water (Milli-Q, Millipore Corp., Bedford, MA, USA) for 60 s (referred to as water treatment). After dipping chopped broccoli, excess water was drained using a plastic mesh. Chopped broccoli non-dipped in water was used as the control to evaluate the effect of 1-MCP when applied alone, whereas chopped broccoli dipped in water was used as the control for samples treated with DPI or PHEN applied alone or in combination with each other or with 1-MCP. Broccoli samples treated with inhibitors and the controls were stored inside hermetic plastic containers in an incubator at 20 °C for 21 h. Samples used to determine the expression of glucosinolate biosynthetic genes and the accumulation of individual GS were collected at 9 h and 21 h of storage, respectively. Immediately after sampling, the tissue was frozen with liquid nitrogen and maintained at −80 °C.

### 3.4. RNA Extraction and Quantitative Real-Time Reverse Transcription-PCR (qRT-PCR)

RNA extraction was performed by the hot borate method [31]. RNA quality and integrity as well as the RNA integrity number (RIN) were assessed as previously reported [3]. Total RNA was treated with DNase and cleaned following the procedures reported by Torres-Contreras et al. [3]. The quality of total RNA was determined according to the following parameters: OD 260/280 > 1.9 and OD 260/230 > 1.5. Three independent RNA extractions of all samples were performed. cDNA was synthesized from RNA extracted from all samples [3]. The qRT-PCR analysis was performed in a Gene 3000 Rotor System (Corbett Life Science, San Francisco, CA, USA) using a 36-well rotor and the Brilliant III Ultra-Fast SYBR Green qPCR Master Mix (Agilent Technologies, Santa Clara, CA, USA), and following the conditions, procedures, and analysis previously described by Salzman et al. [32] Primers were designed using the Primer Premier 5.0 software (Premier Biosoft International) (Supplementary Materials, Table S2). The qRT-PCR analysis was performed with three biological replicates and three technical replicates for each gene validated (*n* = 9). Amplification specificity of each set of primers was determined by analysis of the cleavage curve and amplicon size on agarose gel electrophoresis, to ensure absence of non-specific PCR products. Differential gene expression was calculated using the 2^−ΔΔCt^ method following the protocol of Livak et al. [33] and *actin 2* as a housekeeping gene [3], where:

$$△△\mathrm{Ct}=\left(△{\mathrm{CT}}_{\mathrm{samples}\;\mathrm{before}\;\mathrm{storage}\;\mathrm{cDNA}}\right)-\left(△{\mathrm{CT}}_{\mathrm{samples}\;\mathrm{after}\;\mathrm{storage}\;\mathrm{cDNA}}\right)$$

$$△△\mathrm{Ct}={(\mathrm{mean}\;\mathrm{CT}\;\mathrm{cDNA}}_{\mathrm{test}\;\mathrm{primers}}\;)\;-{\;(\mathrm{mean}\;\mathrm{CT}\;\mathrm{cDNA}}_{\mathrm{actin}\;2\;\mathrm{primers}})$$

### 3.5. Glucosinolate Analysis

The glucosinolate extraction, desulfation, and analysis were performed as previously described [1,2,30,34]. Briefly, 10 mL of methanol:water (70:30, *v*:*v*) at 70 °C was homogenized with 0.2 g of freeze-dried broccoli, followed by the addition of 50 μL of the internal standard (sinigrin solution, 3 mM). Samples were incubated (70 °C for 30 min), and the extracts were tempered at room temperature, followed by centrifugation (3000× *g*, 5 min, 4 °C). GS were desulphated and purified as previously detailed [30]. Desulfoglucosinolates were identified and quantified by HPLC- diode array detector (DAD) and HPLC-ESI-MS^n^ with the chromatographic methods reported by Moreira-Rodríguez et al. [30]. The HPLC- DAD system used was composed of a quaternary pump, an autosampler, and a DAD (1260 Infinity, Agilent Technologies, Santa Clara, CA, USA). For the HPLC-ESI-MS^n^ analysis, a MS Finnigan LCQ Deca XP Max, Ion trap mass spectrometer coupled at the exit of the DAD and equipped with a Z-spray ESI source, and run by Xcalibur version 1.3 software (Thermo Finnigan-Surveyor San José, CA, USA) was used. Glucosinolate concentrations were reported as mmol of desulfo-glucoraphanin equivalents per kg of broccoli on a dry weight (DW) basis.

### 3.6. Statistical Analysis

Statistical analyses were conducted with three biological replicates. Data presented correspond to the mean values of treatments with their standard error. Analyses of variance (ANOVA) were performed with the JMP software version 9.0 (SAS Institute Inc., Cary, NC, USA) and mean separations using the LSD test (*p* < 0.05).

## Figures and Tables

**Table 1:** Individual glucosinolate concentration in broccoli chops treated with different concentration of signaling molecule inhibitors and stored for 21 h at 20 °C.

Treatment	Glucosinolate Concentration (μmol/Kg DW) ^abc^											
	Glucoraphanin		4-Hydroxyglucobrassicin		Glucobrassicin		4-Metoxyglucobrassicin		Neoglucobrassicin		Total	
0 h	10502.5 ± 748.1	def	1670.2 ± 93.7	hi	8157.7 ± 615.7	bc	1914.9 ± 122.9	f	4207.1 ± 579.5	a	22245.4 ± 1472.8	efg
0 h water	10129.6 ± 1140.2	ef	1180.7 ± 165.4	i	7273.5 ± 556.6	cde	1518.9 ± 112.8	gh	3337.7 ± 485.1	ab	20102.8 ± 1669.0	fg
Control	13255.3 ± 104.9	b	4423.6 ± 373.5	a	9425.1 ± 604.4	a	2503.2 ± 153.7	cde	4010.7 ± 272.1	a	29607.2 ± 1214.5	a
Water	11443.9 ± 454.0	cde	4194.9 ± 92.8	ab	7367.8 ± 114.5	cd	2881.7 ± 3.2	ab	3703.8 ± 284.9	ab	25887.4 ± 358.7	bc
1-MCP 500ppb	12813.7 ± 322.6	bc	4158.9 ± 106.4	abc	8470.9 ± 188.0	ab	2628.0 ± 63.7	abcd	1679.8 ± 386.4	c	28071.5 ± 635.1	ab
1-MCP 1000 ppb	15115.9 ± 835.7	a	3681.9 ± 141.2	bcde	8802.3 ± 291.8	ab	1734.8 ± 44.5	fg	2306.5 ± 589.6	b	29334.9 ± 814.8	a
1-MCP 2000 ppb	8964.4 ± 336.8	fg	1250.4 ± 63.5	i	7204.1 ± 35.4	cde	2451.3 ± 14.0	cde	3159.7 ± 666.3	abc	22583.1 ± 356.6	def
DPI 3.17 μM	12041.6 ± 95.9	bcd	3348.9 ± 103.6	de	6729.9 ± 38.1	de	2587.2 ± 34.4	bcd	3458.4 ± 232.1	ab	24707.6 ± 233.1	cde
DPI 31.7 μM	9941.5 ± 126.5	ef	2223.1 ± 163.4	gh	6734.9 ± 146.5	de	2266.9 ± 46.2	e	2650.9 ± 929.9	abc	21166.4 ± 220.9	fg
DPI 317 μM	15843.6 ± 538.4	a	1100.4 ± 33.3	i	7227.0 ± 131.4	cde	1417.9 ± 32.4	h	3160.8 ± 468.3	abc	25588.9 ± 658.6	def
PHEN 0.1 mM	13131.7 ± 625.2	b	3505.8 ± 422.0	cde	6181.7 ± 329.6	ef	2751.2 ± 183.8	abc	2272.1 ± 235.9	bc	25570.5 ± 1431.5	bcd
PHEN 1.0 mM	8220.8 ± 436.8	g	3038.1 ± 246.6	ef	5624.4 ± 186.7	f	2383.8 ± 110.5	de	3255.6 ± 281.6	abc	19267.1 ± 888.9	g
PHEN 10 mM	15843.5 ± 538.3	a	2569.4 ± 77.9	fg	4479.5 ± 77.4	g	2903.8 ± 26.3	a	3232.7 ± 257.8	abc	25144.9 ± 356.2	bcde

Measurements were taken after 21 h of storage at 20 °C, except for the 0 h control sampling. ^a^ Concentrations were determined based on dry weight basis. ^b^ Data represent the mean of 3 replicates ± standard error of the mean. ^c^ Columns with different letters (a, b, c, d, e, f) indicate statistical difference by the LSD test (*p* ≤ 0.05). Abbreviations: 1-methylcyclopropene (1-MCP); diphenyleneiodonium chloride (DPI), and phenidone (PHEN).

## Data Availability

The data presented in this study are available within the article and its Supplementary Materials.

## Supplementary Materials

The following are available online at https://www.mdpi.com/article/10.3390/plants10122660/s1, Figure S1: TypicalHPLC-DAD chromatogram of desulfoglucosinolates (shown at 227 nm) obtained from broccoli extracts, Table S1: Individual glucosinolate concentration in broccoli chops during 24 h of storage at 20 °C, Table S2. Primers used in qRT-PCR to evaluate the expression of genes related with the biosynthesis of glucosinolates in in broccoli.
