# Supplementary material for: Chemical Genetics Applied to Elucidate the Physiological Role of Stress-Signaling Molecules on the Wound-Induced Accumulation of Glucosinolates in Broccoli
Source: Plants (Basel). 2021 Dec 3;10(12):2660. doi: 10.3390/plants10122660 (PMC8706940; doi:10.3390/plants10122660)
Supplement: Supplementary file 1 [file plants-10-02660-s001.zip › plants-1477781-supplementary.pdf]

plants

MDPI

Supplementary Material

Chemical Genetics Applied to Elucidate the Physiological Role of Stress Signaling Molecules on the Wound-Induced Accumulation of Glucosinolates in Broccoli

Ana M. Torres-Contreras [1], Vimal Nair [2], Carolina Senés-Guerrero [3], Adriana Pacheco [1], Mauricio González-Agüero [4], Perla A. Ramos-Parra [1], Luis Cisneros-Zevallos [2] and Daniel A. Jacobo-Velázquez [3,*]

[1] Tecnologico de Monterrey, Escuela de Ingeniería y Ciencias, Av. Eugenio Garza Sada 2501, Monterrey, NL C.P. 64849, México; marieltorres2811@gmail.com (A.M.T.-C.); carolina.senes@tec.mx (C.S.-G.); adrianap@tec.mx (A.P.); perlaramos@tec.mx (P.A.R.-P.)

[2] Texas A&M University, Department of Horticultural Sciences, College Station, TX, 77843, USA; vimal.nair16@gmail.com (V.N.); lcisnero@tamu.edu (L.C.-Z.)

[3] Tecnologico de Monterrey, Escuela de Ingeniería y Ciencias, Av. General Ramón Corona 2514, Zapopan, Jal. C.P. 45201, México

[4] Institute for Agricultural Research, INIA-La Platina, Postharvest Unit. Santa Rosa 11610, Santiago, Chile; maugonza@gmail.com

* Correspondence: djacobov@tec.mx; Tel.: +52-312-119-1650

Citation: Torres-Contreras, A.M.; Nair, V.; Senés-Guerrero, C.; Pacheco, A.; González-Agüero, M.; Ramos-Parra, P.A.; Cisneros-Zevallos, L.; Jacobo-Velázquez, D.A. Chemical Genetics Applied to Elucidate the Physiological Role of Stress Signaling Molecules on the Wound-Induced Accumulation of Glucosinolates in Broccoli. *Plants* **2021**, *10*, 2660. https://doi.org/10.3390/plants10122660

Academic Editor: Maria Elena Cartea

Received: 8 November 2021
Accepted: 1 December 2021
Published: 3 December 2021

Publisher's Note: MDPI stays neutral with regard to jurisdictional claims in published maps and institutional affiliations.

Copyright: © 2021 by the authors. Licensee MDPI, Basel, Switzerland. This article is an open access article distributed under the terms and conditions of the Creative Commons Attribution (CC BY) license (https://creativecommons.org/licenses/by/4.0/).

Determination of the optimum storage time for the accumulation of glucosinolates (GS) during storage of wounded-broccoli

In order to find the optimum storage time for the accumulation of glucosinolates (GS) in broccoli, their content was evaluated every 3 h during 24 h storage (at 20 ºC) in broccoli chops (**Table S1**). Except for glucoerucin, all individual GS showed the highest accumulation at 21 h of storage. At this storage time, 4-hydroxyglucobrassicin and glucobrassicin showed 186 and 185% higher content, respectively, as compared with chops before storage (time 0 h). Furthermore, neoglucobrassicin and glucoraphanin increased 40% as compared with time 0 h samples. According with these results, 21 h was selected as the storage time (at 20 ºC) of broccoli chops to perform he following experiments focused on understanding the role of signaling molecules on the wound-induced accumulation of GS and phenolic compounds (PC) in broccoli.

Table S1. Individual glucosinolate concentration in broccoli chops during 24 h of storage at 20ºC.

Storage time (h)	Glucosinolate concentration (μmol/Kg DW)[abc]											
	GRA		4HGBS		GE		GBS		NGB		Total	
**0**	1426.3±92.5	d	641.1±62.5	f	11965.8±1122.4	d	911.3±87.9	f	1776.5±183.5	d	15294.8±1408.9	cd
**1**	1906.8±167.7	abc	819.0±65.2	ef	15389.1±1144.8	b	997.4±96.3	ef	2653.7±223.9	ab	19859.2±1517.2	ab
**3**	1617.3±167.7	bcd	880.6±55.9	ef	15177.5±1308.9	bc	1223.1±23.5	cde	1906.4±274.5	cd	19187.6±1655.9	bc
**6**	2039.3±197.9	a	1063.2±63.1	cde	18327.3±628.7	a	1270.2±79.1	cd	2783.7±99.5	a	23445.0±1039.8	a
**9**	1796.4±29.5	abc	937.2±89.0	def	11837.3±1.2	d	935.8±27.9	f	1878.7±35.2	cd	15588.9±631.7	cd
**12**	1946.5±98.4	ab	1182.8±139.1	bcd	14200.2±1271.9	bcd	1086.4±102.6	def	2252.4±347.7	abcd	18721.7±1853.2	bc
**15**	1996.1±98.4	a	1240.9±126.8	bc	12412.6±544.4	cd	1085.2±65.9	def	2217.6±112.9	bcd	16956.3±811.7	bc
**18**	1633.7±40.2	bcd	1423.2±94.4	b	11938.5±1192.3	d	1407.3±143.3	bc	1947.8±150.9	cd	16716.8±1524.6	bcd
**21**	1973.3±89.9	a	1830.4±144.8	a	12252.8±861.3	d	1703.4±118.7	a	2408.1±169.8	abc	18194.6±1286.5	bc
**24**	1579.2±106.2	cd	1318.5±39.4	bc	8159.8±565.5	e	1614.9±80.7	ab	1916.5±83.7	cd	13009.6±685.7	d

[a]Individual glucosinolate concentration is reported in dry weight (DW) basis. [b]Data represents the mean of 3 replicates ± standard error of the mean. [c]Columns with different letters in each wounding intensity treatment indicate statistical difference by the LSD test (p≤0.05). Abbreviations: Glucoraphanin (GRA), 4-hydroxyglucobrassicin (4HGBS), glucoeuricin (GE), glucobrassicin (GBS), neoglucobrassicin (NGBS).

Identification of individual glucosinolates

Individual glucosinolates identified in broccoli included two aliphatic (glucoraphanin and glucoiberin), and four indolic (4-hydroxyglucobrassicin, glucobrassicin, 4-methoxyglucobrassicin and neoglucobrassicin) glucosinolates (**Figure S1**).

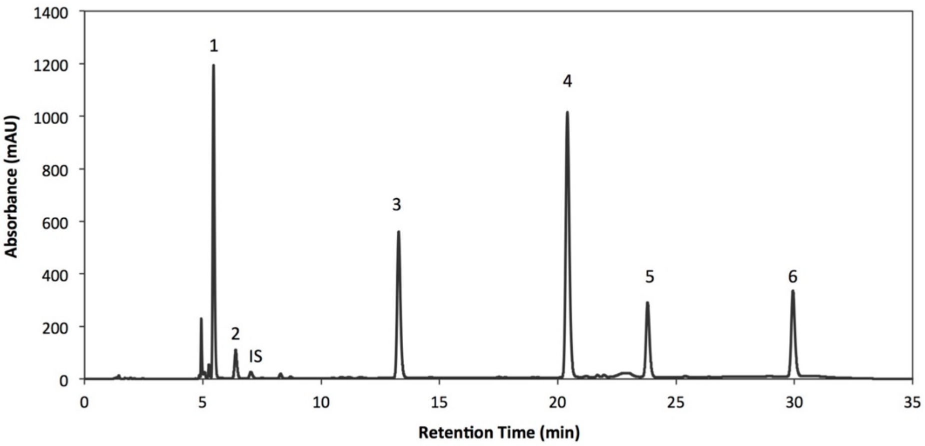

Peak Number (Retention Time, min)	λ Max (nm)	Tentative Identification	$[M-Z]^-$ (*m*/*z*)	MS Fragments [d]
1 (5.3)	222	Desulfoglucoiberin [b,c]	342	**179**, 131
2 (6.6)	222	Desulfoglucoraphanin [a,b,c]	356	**193**
3 (13.4)	222	4-hydroxydesulfoglucobrassicin [b,c]	383	**221**, 203, 153
4 (20.6)	222, 280	Desulfoglucobrassicin [b,c]	367	**204**, 187, 155, 129
5 (23.8)	222, 290	4-methoxydesulfoglucobrassicin [b,c]	397	**234**, 204, 154, 139
6 (30.3)	222, 290	Desulfoneoglucobrassicin [b,c]	397	**234**, 204, 154, 129

Figure S1. Typical HPLC-DAD chromatogram of desulfoglucosinolates (shown at 227 nm) obtained from broccoli extracts. Identification of individual glucosinolates was obtained by HPLC-DAD and HPLC-ESI-MS^n. Identification was obtained by HPLC-DAD and HPLC-ESI-MS^n. [a]Identified based on their spectra characteristics and their ratio as compared with authentic standards. [b]Identified based on their spectra characteristics and order of elution as compared with previous reports (Villarreal-García et al, 2016; Torres-Contreras et al., 2017). [c]Identified based on their spectra characteristics and their mass-to-charge ratio as compared with a previous report (Vallejo et al., 2003). [d]Major fragment ions are shown in bold.

Table S2. Primers used in qRT-PCR to evaluate the expression of genes related with the biosynthesis of glucosinolates in in broccoli.

Gene	Description according GenBank	Forward primer (5´-3´)	Reverse primer (5´-3´)	Amplicon size (bp)
BoCYP79B2	cytochrome P450 79B2	GGAATGGTCCCAACGATGCTAA	AGAGCGTCTTGTTGCTTGAGTA	167
BoST5a	cytosolic sulfotransferase 16	TCTCCCAACAATCCCATTCC	TGGAAGGGAAGCGATGAAGT	166
BoST5b	cytosolic sulfotransferase 18	GGTGCCTGTCTTTGGGTAGC	TGGTCACTGGTGGGTACAGC	109
BoST5c	cytosolic sulfotransferase 17	GAGGGTCGTGAAGCTTTGTAG	GCGTCAAATAATTCTCCCAGTC	149
BoIGMT1	indole glucosinolate O-methyltransferase 1	CATGATTGGACCGACGAAGA	TCTCTTTTCCACCGGAGCAT	187
BoMYB122	transcription factor MYB122	TAAGCTCATCGCCTACGTCCAA	GCTCCTCTTCGCTAAACTCACC	155
BoACT2	Actin 2	GTCGCTATTCAAGCTGTTCTCT	GAGAGCTTCTCCTTGATGTCTC	251

Abbreviations: *Brassica oleracea* (Bo).

References:

Villarreal-García, D.; Nair, V.; Cisneros-Zevallos, L; Jacobo-Velázquez, D.A. Plants as biofactories: Postharvest stress-induced accumulation of phenolic compounds and glucosinolates in broccoli subjected to wounding stress and exogenous phytohormones. *Front. Plant Sci.* **2016**, *7*, 45. https://doi.org/10.3389/fpls.2016.00045

Torres-Contreras, A.M.; Nair, V.; Cisneros-Zevallos, L.; Jacobo-Velázquez, D.A. Stability of bioactive compounds in broccoli as affected by cutting styles and storage time. *Molecules* **2017**, *22*, 636. https://doi.org/10.3390/molecules22040636

Vallejo, F.; Tomás-Barberán, F.A.; Benavente-García, A.G.; García-Viguera, C. Total and individual glucosinolate contents in inflorescences of eight broccoli cultivars grown under various climatic and fertilisation conditions. *J. Sci. Food Agric.* **2003**, *83*, 307–313. https://doi.org/10.1002/jsfa.1320
